# Efficacy and safety of first- versus second-generation fibroblast growth factor receptor (FGFR) inhibitors in advanced or metastatic urothelial carcinoma with FGFR alterations: a systematic review and meta-analysis

**DOI:** 10.3389/fphar.2026.1850131

**Published:** 2026-09-30

**Authors:** Zhe Wang, Bo Shao, Danxue Huang, Liyuan Ke

**Affiliations:** Cancer Hospital of Dalian University of Technology, Liaoning Cancer Hospital and Institute, Shenyang, China

**Keywords:** efficacy, FGFR inhibitors, meta-analysis, safety, systematic review, urothelial carcinoma

## Abstract

**Introduction:**

We reviewed the clinical studies on fibroblast growth factor receptor (FGFR) inhibitors for the treatment of urothelial carcinoma to conduct the meta-analysis of clinical efficacy and safety, aiming to provide more precise treatment options for patients with FGFR alterations.

**Method:**

We performed the literature search and screening, then calculated and analyzed using the STATA 14.0 software and Review Manager 5.3 software.

**Results:**

A total of 1528 patients from seventeen studies focusing on seven different FGFR inhibitors were collected. The efficacy analysis indicated the pooled objective response rate (ORR) was 32% (95% CI 26% to 39%) and the pooled disease control rate (DCR) was 64% (95% CI 53% to 75%) for patients with FGFR alterations treated with FGFR inhibitors. The pooled ORR and DCR of second-generation FGFR inhibitors were both significantly higher than those of first-generation FGFR inhibitors (ORR: 0.35, 95% CI 0.29 to 0.41 versus 0.07, 95% CI 0.02 to 0.16, p < 0.001; DCR: 0.68, 95% CI 0.61 to 0.75 versus 0.27, 95% CI 0.16 to 0.40, p < 0.001). The safety analysis indicated any-grade TRAEs were comparable between first- and second-generation FGFR inhibitors, but grade ≥3 TRAEs were significantly less frequent with second-generation FGFR inhibitors versus first-generation FGFR inhibitors (0.49, 95% CI 0.40 to 0.57 versus 0.67, 95% CI 0.57 to 0.76; p = 0.006). The common target toxicities associated with FGFR inhibitors, such as hyperphosphatemia, stomatitis and PPES, were more frequently occurred in second-generation FGFR inhibitors compared to first-generation FGFR inhibitors.

**Conclusion:**

The second-generation FGFR inhibitors have advantages in both efficacy and safety, but special adverse reactions still require additional attention.

## 1 Introduction

Urothelial carcinoma (UC) is a common type of malignant tumor, which is further classified into upper urinary tract carcinoma and lower urinary tract carcinoma. The predominant type is bladder cancer, and its incidence ranks 10th in the global cancer statistics 2020 (Sung et al., 2021). For patients with advanced or metastatic UC, systemic chemotherapy remained an effective treatment option (von der Maase et al., 2000; Sternberg et al., 2006). Nevertheless, more than 50% of patients with metastatic UC are not suitable for cisplatin treatment (De Santis et al., 2012). Even if patients successfully undergo platinum-based chemotherapy, their progression-free survival (PFS) is usually several months (Loehrer et al., 1992). Immune checkpoint inhibitors have been proven to be effective for patients who are not suitable for cisplatin treatment, but only approximately 30% of patients with metastatic UC respond to immune checkpoint inhibitors (Suzman et al., 2019; Lopez-Beltran et al., 2021). The approval of the fibroblast growth factor receptor (FGFR) protein-tyrosine kinase inhibitor erdafitinib has ushered in a new era of targeted treatment for urothelial carcinoma (Roskoski, 2020).

Approximately 20% of advanced or metastatic UC exhibit FGFR alterations, and this rate is as high as 36% in upper urothelial carcinomas (Murugesan et al., 2022). FGFR1–4 is a family of receptor tyrosine kinases, genetic alterations in which promote to the proliferation of tumor cells (Murugesan et al., 2022). Some small-molecule FGFR inhibitors have been developed for the treatment of various cancer patients with FGFR alterations (Bahleda et al., 2019; Ardizzone et al., 2020; Navid et al., 2020; Vogel et al., 2023). Binding to the ATP-binding site of the tyrosine kinase domain, FGFR inhibitors are usually classified as first-generation FGFR inhibitors (multi-kinase type FGFR inhibitors) or second-generation FGFR inhibitors (selective FGFR inhibitors). Currently, among first-generation FGFR inhibitors, only derazantinib and dovitinib were developed for the treatment of urothelial carcinoma. Second-generation FGFR inhibitors can further be classified as pan-FGFR inhibitors (erdafitinib, futibatinib, rogaratinib, and resigratinib), as well as FGFR1/2/3 inhibitors (pemigatinib, infigratinib, fexagratinib, and zoligratinib) (Katoh et al., 2024). According to the mechanism of drug-target interaction, FGFR inhibitors can also be classified into non-covalent inhibitors that inhibit the enzymatic activity of FGFR through ATP competition and covalent inhibitors that interact with the specific cysteine residues in the ATP-binding pocket (Dai et al., 2019).

As the first approved FGFR inhibitor, erdafitinib is indicated for the second or later line treatment of advanced or metastatic urothelial carcinoma patients with FGFR3 genetic alteration. The phase 2 study enrolled patients with advanced or metastatic UC who had received chemotherapy but experienced disease progression or were not suitable for chemotherapy (Loriot et al., 2019). After confirming the presence of FGFR gene alterations, they were treated with erdafitinib, and the objective response rate (ORR) was 40% [95% confidence interval (CI) 30 to 49)] (Siefker-Radtke et al., 2022). The phase 3 THOR study was divided into two cohorts to assess the efficacy of erdafitinib compared with chemotherapy or immunotherapy (Loriot et al., 2023; Siefker-Radtke et al., 2024). The results showed that erdafitinib provided greater survival benefits than chemotherapy, but this advantage was not observed when compared with immunotherapy (Loriot et al., 2023; Siefker-Radtke et al., 2024). Apart from erdafitinib, there are still many other FGFR inhibitors currently undergoing clinical trials. In the phase II/III study FORT-1, patients with FGFR1/3 alternations and ≥1 prior platinum-containing regimen achieved ORR 20.7% (95% CI 12.7 to 30.7) after being treated with rogaratinib, but no additional survival benefits for them were achieved compared with chemotherapy (Sternberg et al., 2023). Other FGFR inhibitors, infigratinib, pemigatinib, and futibatinib, approved for cholangiocarcinoma, are also undergoing clinical studies for UC.

Safety and tolerability are the key considerations for all targeted FGFR drugs, as FGFR is expressed in many different cell types and regulates a variety of physiological processes. The common target toxicities associated with FGFR inhibitors include hyperphosphatemia, nail disorders, stomatitis, palmar-plantar erythrodysesthesia syndrome (PPES), rash, and retinal disorders (Subbiah and Verstovsek, 2023). A thorough understanding of the characteristics of treatment-related adverse events (TRAEs) caused by FGFR inhibitors is crucial for optimizing their therapeutic efficacy while maintaining patient safety and compliance. In order to provide treatment options for patients with advanced or metastatic UC who have genetic alterations in the FGFR gene, we conducted a systematic review and meta-analysis study to evaluate the efficacy and safety of FGFR inhibitors comprehensively.

## 2 Methods

### 2.1 Search strategy

Two members of the research team independently and systematically searched PubMed, Embase, and Web of Science databases to retrieve relevant literature published before July 10, 2025. The search strategy is recorded in Supplementary Table S1. Meanwhile, we conducted a manual search on ASCO, ESMO, ClinicalTrial.gov, and Cochrane Library.

### 2.2 Selection criteria

We formulated the following inclusion and exclusion criteria. Inclusion criteria: (1) The patients were diagnosed with advanced or metastatic UC. (2) The patients received treatment with an FGFR inhibitor. (3) The patients were required to have one or more FGFR alterations. (4) The outcomes included ORR, disease control rate (DCR), overall survival (OS), PFS, and adverse events (AEs). (5) The types of studies include phase I, phase II, and phase III clinical trials as well as real-world studies. The design of the trials can be either randomized controlled trials or single-arm trials. Exclusion criteria: Reviews, case reports, animal studies, and non-English articles.

### 2.3 Data extraction and risk of bias

Data extraction consisted of four parts, including literature information, trial characteristics, participants’ baseline, and outcomes. The literature information recorded the first author and the publication year as the identification markers for the literature. The characteristics of the trial include clinical trial registration number, clinical stage, region, number of participants, trial drug, and its dosage. Meanwhile, we also collected the participants’ gender, age, ECOG score, primary tumor location, FGFR mutation status, previous lines of systemic therapy, and outcomes.

We used the Cochrane tool to assess the quality of randomized controlled trials and employed the Review Manager 5.3 software to draw the risk of bias diagram (Higgins et al., 2011). We used the MINORS tool to conduct quality assessment of non-randomized trials and drew the scoring table (Slim et al., 2003).

### 2.4 Statistical methods and data analysis

The efficacy analysis focused on OS, PFS, and ORR. The safety analysis included any grade TRAEs, grade ≥3 TRAEs, AEs leading to treatment discontinuation, and common TRAEs. The heterogeneity of the included studies was measured by the *p*-value of Cochrane’s Q test and Higgins’ *I* ^*2*^ statistic. Since there was no significant heterogeneity (*p*-value > 0.1 or *I* ^*2*^ < 50%), the fixed-effect model was adopted; otherwise, the random-effect model was used (Higgins et al., 2003). Sensitivity analysis was used to evaluate the impact of each study on the stability of the results. Publication bias was represented by a funnel plot. Egger’s test and Begg’s test were used to quantify the funnel plot, with *p* value > 0.05 indicating the absence of publication bias (Egger et al., 1997; Begg and Mazumdar, 1994). All the data analyses were conducted via the Stata 14.0 software.

## 3 Results

### 3.1 Search and selection

We conducted literature searches in the database by referring to the search terms. After four steps of the screening process, we finally selected seventeen studies on the treatment of UC with FGFR inhibitors. The process of literature search and screening was independently completed by two researchers. In case of any disputes, a third researcher would make the final judgment. The flow diagram for the selection of included studies is shown in Supplementary Figure S1.

### 3.2 Characteristic of included studies

The characteristics of included studies are listed in Table 1. The included seventeen studies reported a total of seven types of FGFR inhibitors, including erdafitinib, rogaratinib, pemigatinib, futibatinib, infigratinib, dovitinib, and derazantinib. In the aforementioned study, nine studies on erdafitinib reported the clinical research results of phase I, phase II, phase III, as well as real-world studies. Three studies on the rogaratinib released the results of its clinical trials from phase I, phase II, and phase III. Only the phase II studies were reported for the other five FGFR inhibitors: pemigatinib, futibatinib, infigratinib, dovitinib, and derazantinib. Four real-world studies were conducted in the United States, Israel, and Brazil, while all other clinical studies were carried out globally. A total of 1528 patients were involved, and their clinical characteristics are detailed in the Supplementary Table S2.

**TABLE 1:** The characteristic of included studies.

Study ID	Trial phase	ClinicalTrials identifier	Region	Sample size	Intervention	Dosage	Reported outcomes
Loriot 2023 (Loriot et al., 2023)	III	NCT03390504	global	266	erdafitinib	8 mg	ORR, PFS, OS, AE
Siefker-Radtke2023 (III) (Siefker-Radtke et al., 2024)	III	NCT03390504	global	351	erdafitinib	8 mg	ORR, DCR, PFS, OS, AE
Siefker-Radtke2022 (Siefker-Radtke et al., 2022)	II	NCT02365597	global	101	erdafitinib	8 mg	ORR, DCR, AE
Siefker-Radtke2023 (II) (Siefker-Radtke et al., 2023)	II	NCT03473743	global	43	erdafitinib	8/9 mg	ORR, DCR
Soria 2016 (Soria et al., 2016)	I	NCT01703481	global	23	erdafitinib	2/9/10/12 mg	ORR
Jiang 2024 (Jiang et al., 2024)	Real-world analysis	—	US	37	erdafitinib	NA	ORR
Rouvinov 2023 (Rouvinov et al., 2023)	Real-world analysis	—	Israel	25	erdafitinib	8 mg	ORR, AE
Monteiro 2021 (Monteiro et al., 2021)	Real-world analysis	—	Brazil	12	erdafitinib	8 mg	ORR, AE
Guercio 2021 (Guercio et al., 2023)	Real-world analysis	—	US	32	erdafitinib	NA	ORR, AE
Sternberg 2022 (Sternberg et al., 2023)	II/III	NCT03410693	global	87	rogaratinib	800 mg	ORR, PFS, OS, AE
Sweis 2024 (Sweis et al., 2024)	Ib	NCT03473756	global	37	rogaratinib + atezolizumab	600/800 mg	ORR, DCR, AE
Joerger 2018 (Joerger et al., 2018)	Ib	NCT01976741	NA	51	rogaratinib	800 mg	ORR, DCR
Necchi 2023 (Necchi et al., 2024a)	II	NCT02872714	global	260	pemigatinib	13.5 mg	ORR, DCR, AE
Koshkin 2024 (Koshkin et al., 2024)	II	NCT04601857	global	43	futibatinib + pembrolizumab	20 mg	ORR, AE
Pal 2018 (Pal et al., 2018)	II	NCT01004224	global	67	infigratinib	125 mg	ORR, DCR, AE
Milowsky 2014 (Milowsky et al., 2014)	II	NCT00790426	global	44	dovitinib	500 mg	ORR, DCR, AE
Necchi 2024 (Necchi et al., 2024b)	Ib/II	NCT04045613	global	49	derazantinib	300/200 mg	ORR, DCR, AE

### 3.3 Risk of bias

The traffic light plots and bar plots for risk of bias assessment by the Cochrane Collaboration’s tool are presented in Supplementary Figure S2. Since no blinding was conducted in the experimental design, the performance and detection bias related to blinding are at a high-risk level. The MINORS item scores for fourteen non-randomized studies are listed in Supplementary Table S3.

### 3.4 Efficacy

As shown in Figure 1, the pooled ORR of seventeen studies was 0.32 (95% CI 0.26 to 0.39) with high heterogeneity (*I* ^2^ = 77.73%, *p* < 0.01). Among these studies, nine studies reported the results of erdafitinib, and the pooled ORR was 0.41 (95% CI 0.37 to 0.45) with low heterogeneity (*I* ^2^ < 0.01%, *p* = 0.94). The subgroup analysis revealed that the ORR of second-generation FGFR inhibitors was higher than that of first-generation FGFR inhibitors, among which the ORR of pan-FGFR inhibitors was higher than that of FGFR1/2/3 inhibitors (second-generation FGFR inhibitors: 0.35, 95% CI 0.29 to 0.41 versus first-generation FGFR inhibitors: 0.07, 95% CI 0.02 to 0.16; *p* < 0.001). The pooled ORR of erdafitinib monotherapy was superior to that of rogaratinib monotherapy, but was comparable to that of FGFR inhibitors combined with immunotherapy. The differences among the above subgroups were all statistically significant. Based on the subgroup analysis of baseline FGFR alterations, we found that benefits of patients with FGFR 3 alterations was consistent with that of patients with FGFR 1-4 alterations. For patients with FGFR 3 mutation, ORR in the pan-FGFR inhibitors group was higher than that in the FGFR1/2/3 inhibitors group. The results were listed in Tables 2, 3.

**FIGURE 1:**
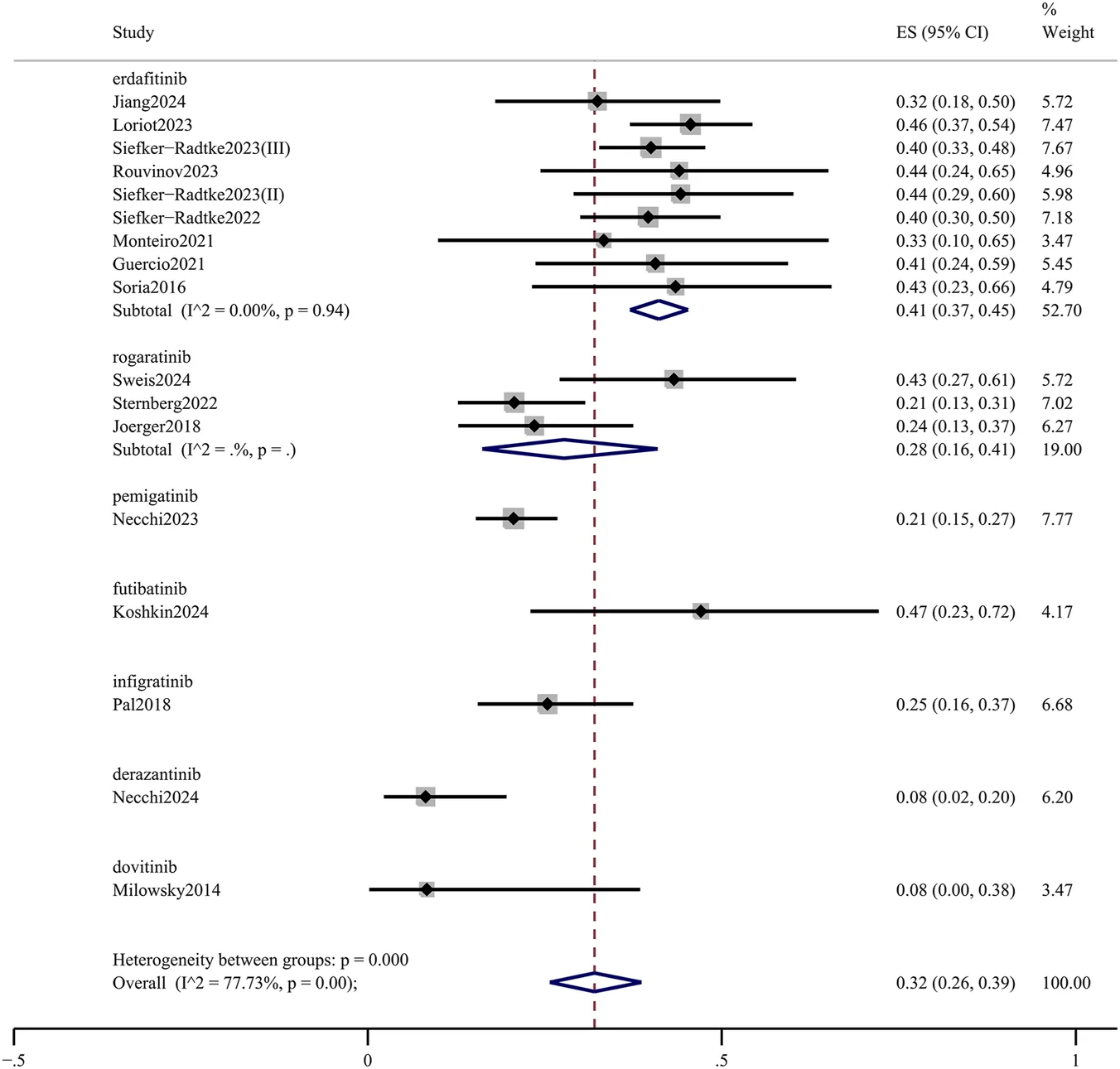
Forest plots for ORR of different FGFR inhibitors.

**TABLE 2:** The subgroup analysis of ORR and DCR in first- and second-generation FGFR inhibitors.

Subgroup	Pooled ORR (95% CI)	Heterogeneity		Significance test *p*	Pooled DCR (95% CI)	Heterogeneity		Significance test *p*
		I^2^	P value			I^2^	P value	
First-/Second-generation FGFR inhibitors								
First-generation FGFR inhibitors	0.07 (95% CI 0.02 to 0.16)	NA	NA	**<0.001**	0.27 (95% CI 0.16 to 0.40)	NA	NA	**<0.001**
Second-generation FGFR inhibitors	0.35 (95% CI 0.29 to 0.41)	71.88%	<0.01		0.68 (95% CI 0.61 to 0.75)	73.95%	<0.01	
Selective FGFR inhibitors								
Pan-FGFR inhibitors	0.37 (95% CI 0.32 to 0.43)	49.87%	0.02	**<0.001**	0.70 (95% CI 0.61 to 0.77)	73.37%	<0.01	0.103
FGFR1/2/3 inhibitors	0.22 (95% CI 0.17 to 0.27)	NA	NA		0.61 (95% CI 0.55 to 0.67)	NA	NA	
Therapeutic regimen								
Erdafitinib monotherapy	0.41 (95% CI 0.37 to 0.45)	0%	0.94	0.642	0.72 (95% CI 0.63 to 0.80)	66.46%	0.01	0.09
Rogaratinib/futibatinib combined with immunotherapy	0.44 (95% CI 0.31 to 0.58)	NA	NA		0.71 (95% CI 0.58 to 0.83)	NA	NA	
Pan-FGFR inhibitors								
Erdafitinib monotherapy	0.41 (95% CI 0.37 to 0.45)	0%	0.94	**<0.001**	0.72 (95% CI 0.63 to 0.80)	66.46%	0.01	**0.041**
Rogaratinib monotherapy	0.22 (95% CI 0.15–0.29)	NA	NA		0.59 (95% CI 0.51 to 0.67)	NA	NA	

The bold format indicates significant differences.

**TABLE 3:** The subgroup analysis of ORR and DCR in baseline FGFR 3 alterations and FGFR1-4 alterations.

Subgroup	Pooled ORR (95% CI)	Heterogeneity		Significance test *p*	Pooled DCR (95% CI)	Heterogeneity		Significance test *p*
		I^2^	P value			I^2^	P value	
Baseline FGFR Alterations								
FGFR 3 alterations	0.36 (95% CI 0.27 to 0.46)	80.46%	<0.01	0.824	0.64 (95% CI 0.55 to 0.73)	69.96%	0.01	0.327
FGFR1-4 alterations	0.35 (95% CI 0.27 to 0.43)	58.84%	0.02		0.71 (95% CI 0.61 to 0.71)	77.31%	0.01	
FGFR 3 alterations								
Pan-FGFR inhibitors	0.42 (95% CI 0.38 to 0.47)	0%	0.82	<0.01	0.64 (95% CI 0.46 to 0.81)	NA	NA	0.755
FGFR1/2/3 inhibitors	0.22 (95% CI 0.17 to 0.27)	NA	NA		0.61 (95% CI 0.55 to 0.67)	NA	NA	
FGFR1-4 alterations								
Pan-FGFR inhibitors	0.35 (95% CI 0.27 to 0.43)	58.84%	0.02	NA	0.71 (95% CI 0.58 to 0.82)	82.91%	<0.01	0.985
FGFR1/2/3 inhibitors	NA	NA	NA		0.71 (95% CI 0.58 to 0.83)	NA	NA	

As shown in Figure 2, the pooled DCR of nine studies was 0.64 (95% CI 0.53 to 0.75) with high heterogeneity (*I* ^2^ = 88.73%, *p* < 0.01). Among these studies, three studies reported the results of erdafitinib, and the pooled DCR was 0.80 (95% CI 0.73 to 0.86). The subgroup analysis revealed that the pooled DCR of second-generation FGFR inhibitors was significantly higher than that of first-generation FGFR inhibitors (second-generation FGFR inhibitors: 0.68, 95% CI 0.61 to 0.75 versus first-generation FGFR inhibitors: 0.27, 95% CI 0.16 to 0.40; *p* < 0.001). Among the second-generation FGFR inhibitors, the pooled DCR of the pan-FGFR inhibitors showed no significant difference from that of the FGFR1/2/3 inhibitors. The pooled DCR of erdafitinib monotherapy was comparable to that of FGFR inhibitors combined with immunotherapy, and superior to that of rogafitinib monotherapy. For patients with different baseline FGFR alterations, there was no significant difference in the pooled DCR when treated with selective FGFR inhibitors. The results were listed in Tables 2, 3.

**FIGURE 2:**
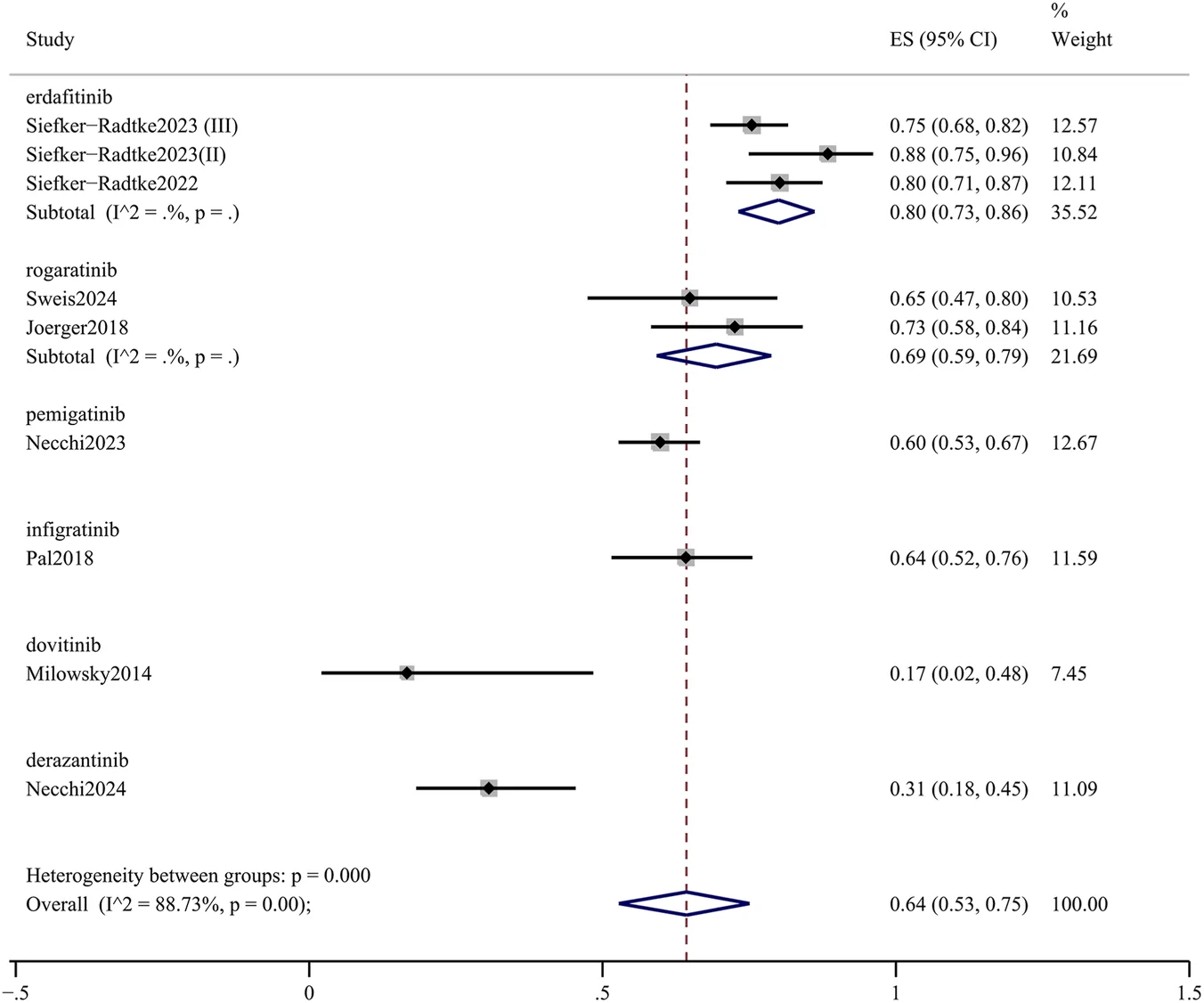
Forest plots for DCR of different FGFR inhibitors.

Three phase III trials respectively investigated the survival benefits of erdafitinib versus chemotherapy, erdafitinib versus immunotherapy, and rogaratinib versus chemotherapy. The pooled hazard ratio for OS in the FGFR inhibitors group versus chemotherapy or immunotherapy was 0.94 (95% CI 0.62 to 1.42) (*p* > 0.05) and for PFS was 0.84 (95% CI 0.57 to 1.24) (*p* > 0.05). The pooled analysis of OS is presented in Supplementary Figure S3, and the pooled analysis of PFS is presented in Supplementary Figure S4. Only the study comparing erdafitinib with chemotherapy demonstrated a significant survival benefit over endatuminib, and the other two studies did not demonstrate a significant advantage of FGFR inhibitors over the control group. The 6-month, 12-month, and 24-month survival rates are detailed in the Supplementary Table S4.

### 3.5 Safety

The forest plots showing TRAEs of any grade and those of grade ≥3 are presented in Figures 3, 4. By summarizing the results of adverse events from twelve studies, the pooled rate of any grade TRAEs was 0.97 (95% CI 0.96 to 0.99) with low heterogeneity (*I* ^2^ = 35.75%, *p* = 0.10). The pooled rate of grade ≥3 TRAEs was 0.52 (95% CI 0.44 to 0.59) with high heterogeneity (*I* ^2^ = 81.51%, *p* < 0.10). As shown in Figure 5, the pooled rate of TRAEs leading to treatment discontinuation was 0.15 (95% CI 0.12 to 0.19) with low heterogeneity (*I* ^2^ = 16.98%, *p* = 0.29). The subgroup analysis showed that there was no significant difference in the pooled rate of any grade TRAEs between the second-generation FGFR inhibitors and the first-generation FGFR inhibitors. However, the pooled rate of grade ≥3 TRAEs in the second-generation FGFR inhibitors was less than that in the first-generation FGFR inhibitors (second-generation FGFR inhibitors: 0.49, 95% CI 0.40 to 0.57 versus first-generation FGFR inhibitors: 0.67, 95% CI 0.57 to 0.76; *p* = 0.006). Detailed data is recorded in Supplementary Tables S5, S6.

**FIGURE 3:**
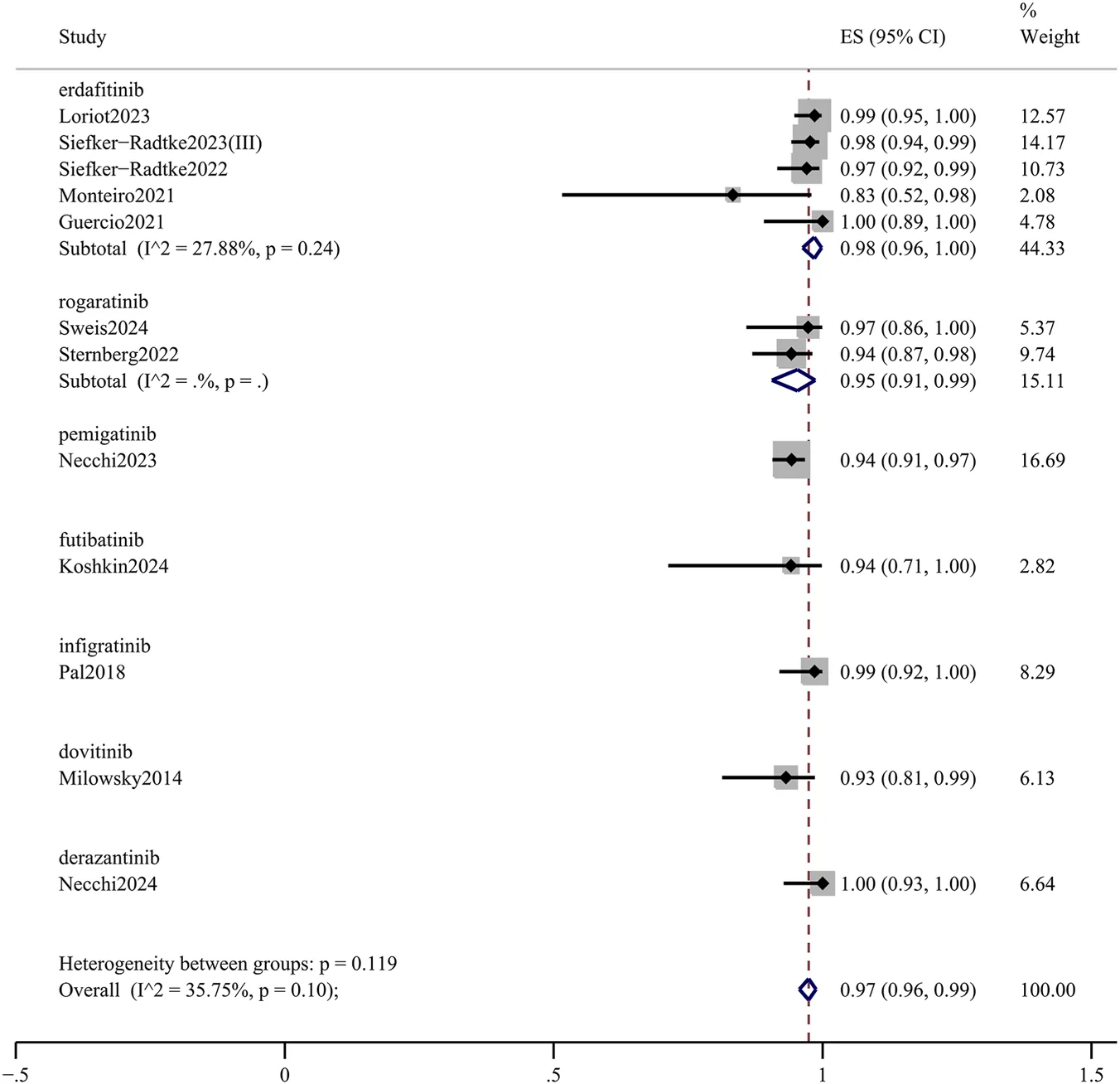
Forest plots for incidence of any grade TRAEs.

**FIGURE 4:**
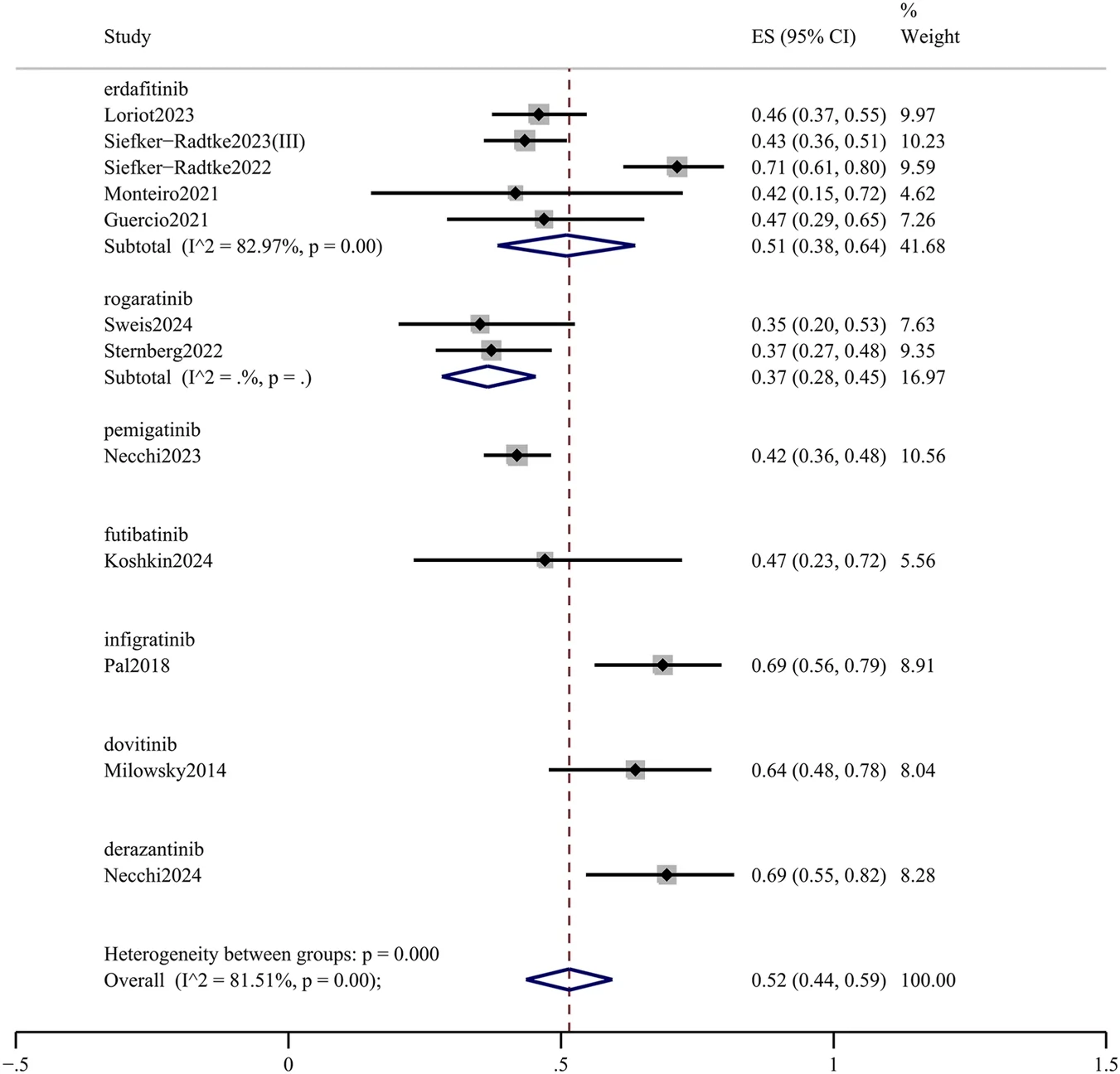
Forest plots for incidence of grade ≥3 TRAEs.

**FIGURE 5:**
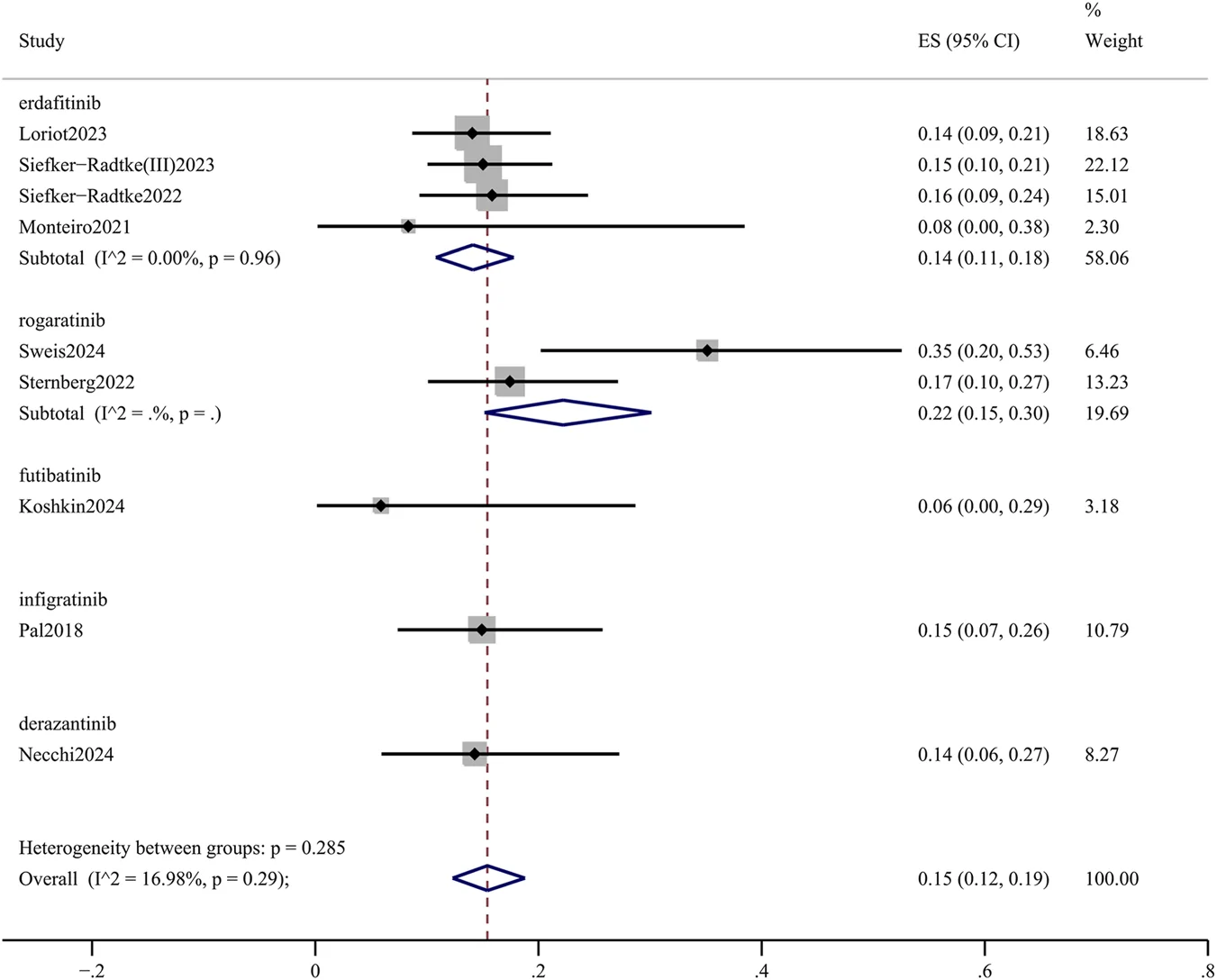
Forest plots for incidence of adverse events leading to treatment discontinuation.

The common TRAEs of FGFR inhibitors were hyperphosphatemia, diarrhea, stomatitis, dry mouth, PPES, constipation, dysgeusia, alopecia, onycholysis, dry skin, increased alanine aminotransferase, increased aspartate aminotransferase, decreased appetite, nausea, fatigue, anemia, dry eye, asthenia, weight decreased, and vomiting. The pooled incidences of any grade and grade ≥3 common TRAEs are summarized in Figures 6, 7. The highest incidence of any grade TRAEs was hyperphosphatemia (0.56, 95% CI 0.44 to 0.67), and that of grade ≥3 AE was PPES (0.07, 95% CI 0.05 to 0.10). The incidence of any grade hyperphosphatemia was significantly higher in second-generation FGFR inhibitors subgroup compared to first-generation FGFR inhibitors subgroup (second-generation FGFR inhibitors: 0.60, 95% CI 0.49 to 0.70 versus first-generation FGFR inhibitors: 0.06, 95% CI 0.02to 0.12; *p* < 0.001). The incidences of grade ≥3 hyperphosphatemia with futibatinib was 18%, while the rates for the other FGFR inhibitors were all below 2%. The incidences of any grade and grade ≥3 diarrhea were no significant difference. The incidence of any grade stomatitis was significantly higher in second-generation FGFR inhibitors subgroup compared to first-generation FGFR inhibitors subgroup (second-generation FGFR inhibitors: 0.33, 95% CI 0.23 to 0.43 versus first-generation FGFR inhibitors: 0.04, 95% CI 0.00 to 0.14; *p* < 0.001), and simultaneously higher in erdafitinib subgroup than rogaratinib subgroup (erdafitinib: 0.44, 95% CI 0.35 to 0.53 versus rogaratinib: 0.10, 95% CI 0.05 to 0.17; *p* < 0.001). The incidence of grade ≥3 stomatitis was also higher in the second-generation FGFR and erdafitinib. PPES has only been reported in second-generation FGFR inhibitors, and the incidence of any grade and grade ≥3 PPES with erdafitinib were both highest (any grade: 0.25, 95%CI 0.20 to 0.31; grade ≥3: 0.07, 95%CI 0.04 to 0.11). Detailed data is recorded in Supplementary Tables S7–S14.

**FIGURE 6:**
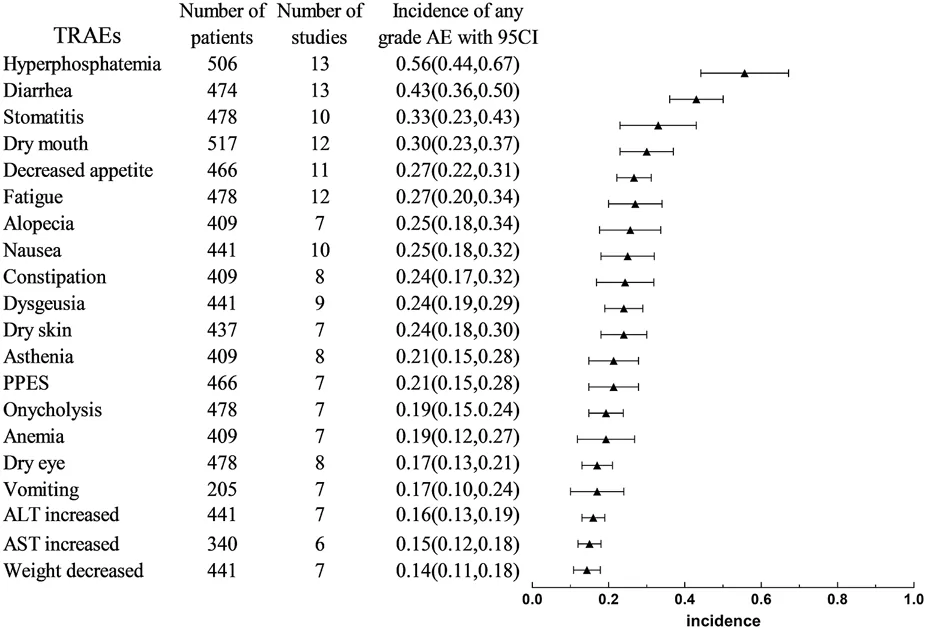
The pooled incidences of any grade common TRAEs in the safety analysis.

**FIGURE 7:**
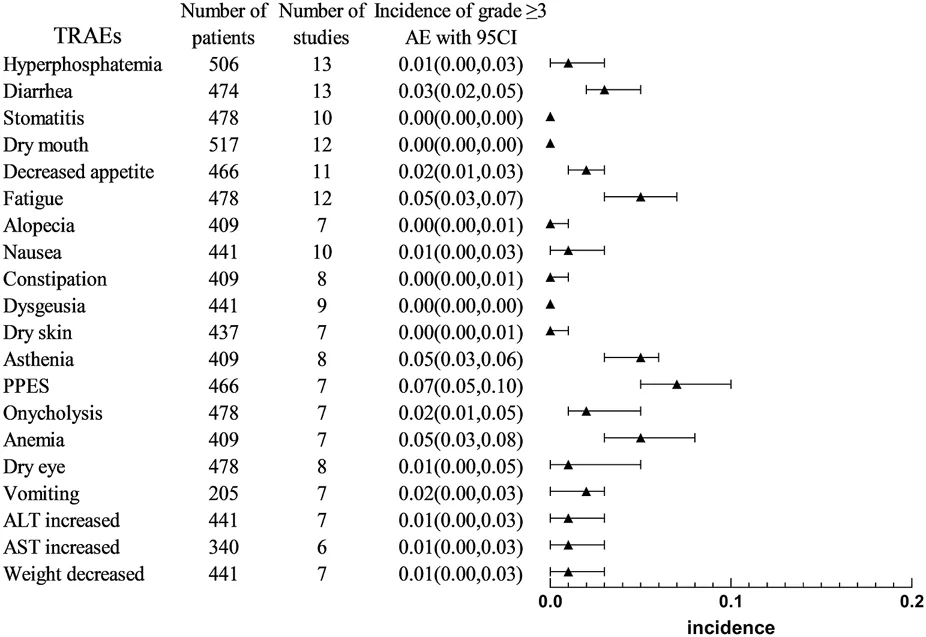
The pooled incidences of grade ≥3 common TRAEs in the safety analysis.

### 3.6 Stability and publication bias

The sensitivity analysis results of ORR, DCR, any grade TRAEs, grade ≥3 TRAEs, and TRAEs leading to treatment discontinuation are exhibited in Supplementary Figures S5–S9. The sensitivity analysis indicated that the removal of any single study had a negligible impact on the results. Thus, the meta-analysis results are stable. Meanwhile, the funnel plots of the four meta-analyses were basically symmetrical, indicating that there was no publication bias (Supplementary Figures S10–S14). The Begg’s test and Egger’s test for publication bias further confirmed the results (Supplementary Table S15).

## 4 Discussion

We conducted the first meta-analysis and systematic review focusing on the efficacy and safety of FGFR inhibitors for advanced or metastatic UC with FGFR alterations. The meta-analysis results were stable, and no publication bias. The result has provided us with some insights and thoughts on FGFR inhibitors for advanced or metastatic UC.

As the FGFR inhibitor approved by the FDA for metastatic UC patients with susceptible FGFR3 genetic alterations, erdafitinib has been studied in multiple clinical trials. In the phase 2 BLC2001 study, ORR was 40% (95% CI 30 to 49) of 101 advanced or metastatic patients with FGFR3 mutation or FGFR2/3 fusion treated with erdafitinib 8 mg/day regimen after at least one course of chemotherapy (Siefker-Radtke et al., 2022). The phase 3 THOR study cohort 2 demonstrated the ORR by investigator assessment was 40.0% in the erdafitinib cohort, which was higher than 21.6% in the pembrolizumab cohort [relative risk (RR) 1.85; 95% CI 1.32 to 2.59] (Siefker-Radtke et al., 2024). In the THOR cohort 1, compared with chemotherapy, the ORR in the erdafitinib group was 45.6% and 11.5% in the chemotherapy group (RR 3.94; 95% CI 2.37 to 6.57), which likewise showed more benefit in the erdafitinib group (Loriot et al., 2023). The subgroup analysis of 27 Japanese in the THOR study cohort 1 showed consistent objective response advantages as observed in the overall population (Matsubara et al., 2024). Inconsistent with the superior results of erdafitinib, the interim results of rogaratinib provided comparable efficacy for advanced UC patients with FGFR1/3 mRNA-positive between the rogaratinib group and the chemotherapy group (Sternberg et al., 2023).

For advanced UC patients who had progression after at least one previous treatment, the comparisons of survival between FGFR inhibitors and chemotherapy or immunotherapy were investigated in three clinical trials. Only the THOR cohort 1 study demonstrated significantly longer OS and PFS in erdafitinib compared with chemotherapy, which provided a novel choice for UC with FGFR alternation after anti-PD-1 OR anti-PD-L1 treatment (Loriot et al., 2023). Although it was confirmed that most patients with FGFR mutations had low PD-L1 expression levels, the low expression of PD-L1 was not associated with poor survival as observed with pembrolizumab in the THOR cohort 2 (Siefker-Radtke et al., 2024). More than that, the pembrolizumab group showed long disease remission and a trend towards OS benefit after 11 months compared to erdafitinib (Siefker-Radtke et al., 2024). After receiving erdafitinib monotherapy, the median OS of patients who received anti-PD-1 or anti-PD-L1 treatment in cohort 1 was longer than that of patients who did not receive anti-PD-1 or anti-PD-L1 treatment in cohort 2 (Siefker-Radtke et al., 2024). The researchers believed that patients in cohort 2 who received erdafitinib as a second-line treatment would have improved median OS compared to those in cohort 1. The FORT-1 study reported the median OS with rogaratinib was 8.3 months in UC patients with high expression of FGFR1/3 mRNA, which was similar to that with infigratinib in UC patients with FGFR3 alterations (Pal et al., 2018).

Although in preclinical UC models, some FGFR inhibitors can inhibit the growth and proliferation of tumor cells, there are still unsatisfactory results in clinical studies. Whether in the FGFR mutation group or the wild group, dovitinib demonstrated limited single-agent activity; thus, it was terminated from the phase 2 clinical trial (Milowsky et al., 2014). The potential reason for unsatisfactory results of dovitinib may be genomic complexity in metastatic UC masking the driving factors of FGFR mutations, unlike the occurrence of FGFR mutations being more common in non-muscle invasive diseases (Milowsky et al., 2014). The objective response rate of UC patients treated with derazantinib was only 8%, which halted further development in metastatic UC (Necchi et al., 2024b). Compared with erdafitinib, the difference in efficacy of derazantinib might be the broader FGFR mutation spectrum expressed by patients enrolled in the trial, as well as the lower dose intensity of derazantinib (Necchi et al., 2024b).

In order to further enhance the therapeutic effect, the combined treatment with FGFR inhibitors and immune checkpoint inhibitors is emerging as a new research direction. The FORT-2 trial was the first study evaluating the efficacy of rogaratinib in combination with atezolizumab in cisplatin-ineligible patients with locally advanced or metastatic UC (Sweis et al., 2024). Compared with the single-agent rogaratinib or atezolizumab, the combined treatment regimen demonstrated a much higher ORR. As the initial treatment for patients with metastatic UC who are platinum ineligible, ORRs of futibatinib plus pembrolizumab were 47% in patients with FGFR alterations and 31% in patients without these alterations (Koshkin et al., 2024). The phase 2 study of futibatinib combined with durvalumab (NCT06263153) is currently recruiting patients with advanced or metastatic UC and those with muscle-invasive bladder cancer who are not suitable for cisplatin treatment. Whether the research results are encouraging and can lead to a new treatment strategy is something that is worthy of our anticipation in the future.

Although the FGFR inhibitors have shown encouraging results, the side effects may limit their clinical application. The most common adverse events include hyperphosphatemia, diarrhea, fatigue, and varied nail, gastrointestinal, dermatologic, or ocular toxicities. As a reference for dose adjustment, hyperphosphatemia is the common adverse event associated with FGFR inhibitors. Hyperphosphatemia is mainly maintained at a level below grade 3 due to management with phosphate-lowering therapies and dietary modification (Subbiah and Verstovsek, 2023). The study evaluating relationships between hyperphosphatemia related to infigratinib and efficacy in advanced or metastatic UC bearing FGFR3 alterations revealed ORR in patients with hyperphosphatemia was 33.3%, which was much higher than that of those without (Lyou et al., 2020). In the phase 2 study of derazantinib, the ORR for patients with metastatic UC and FGFR1-3 genetic aberrations was 8.2%, and the incidence of hyperphosphatemia was also very low, at only 16.3% (Necchi et al., 2024b). The better efficacy must be balanced with the associated toxic effects on the target to optimize the clinical benefits.

Another common adverse event related to FGFR inhibitors was diarrhea, the incidence of which depended on the level of FGFR4 inhibition. FGFR inhibitors induced FGF19/FGFR4/ERK1/2 signaling pathway, altered the balance of bile acids, and thereby affected intestinal peristalsis and the intestinal environment (Wu et al., 2011; Mottacki et al., 2016). The incidence of diarrhea caused by infigratinib was 19%, which was lower than that of the other FGFR inhibitors, which may be related to the limited activity of its FGFR4 receptors (Kommalapati et al., 2021). Eye disorders are special TRAEs of FGFR inhibitors, and central serous retinopathy is one of the adverse events that deserve attention. The incidence of any grade retinal disorders in patients after receiving the rogaratinib treatment was 30%, and the incidence of grade 2 or above was 7% (Sternberg et al., 2023). 42.2% of patients receiving erdafitinib experienced eye disorders other than central serous retinopathy, and the most common ones are dry eye syndrome and conjunctivitis (Loriot et al., 2023). FGFR inhibition affects the downstream signaling pathway of mitogen-activated protein kinase, and its dysregulation is associated with retinal disorders (Francis et al., 2021). Most cases of central serous retinopathy were cured by the end of the trial. Ophthalmologic examinations were necessary during FGFR inhibitors treatment.

The dermatologic toxicities associated with FGFR inhibitors included palmar-plantar erythrodysesthesia syndrome, alopecia, onycholysis, dry skin, stomatitis, and dysgeusia caused by dry mouth. Among these dermatologic toxicities, stomatitis is the first dermatologic toxicity occurring in FGFR inhibitors therapy, with the incidence rate ranging from 8% to 56%. When the patients develop stomatitis, they should maintain good oral hygiene, avoid eating spicy foods, and seek medical attention promptly (Kommalapati et al., 2021). PPES occurred in 25% patients with erdafitinib, 11% patients with rogaratinib, and 12% patients with infigratinib. Notably, the incidence rate of PPES is the highest in ≥ grade 3 TRAEs. In the phase 2 study of erdafitinib, two patients discontinued the medication due to hand-foot syndrome (Siefker-Radtke et al., 2022). The management of PPES involves daily moisturizing care and the application of cortisone creams and topical antibiotics (Subbiah and Verstovsek, 2023). A better understanding of the unique toxicities associated with FGFR inhibitors will help us prevent and effectively manage the adverse events induced by FGFR inhibitors, thereby enhancing their clinical benefits.

## 5 Conclusion

Second-generation FGFR inhibitors have shown effective responses and a low incidence of grade ≥3 TRAEs for advanced UC patients with FGFR alterations compared to first-generation FGFR inhibitors. Erdafitinib, as approved first for UC patients with FGFR alterations, remained an appropriate choice for patients with FGFR alterations in advanced UC treatment strategy for the later stage. The common target toxicities associated with FGFR inhibitors, such as hyperphosphatemia, diarrhea, stomatitis and PPES, still require special attention due to their high incidence in the second-generation FGFR inhibitors.

## Data Availability

The original contributions presented in the study are included in the article/Supplementary Material, further inquiries can be directed to the corresponding author.

## Glossary

AE: adverse events
DCR: disease control rate
FGFR: fibroblast growth factor receptor
ORR: objective response rate
OS: overall survival
PFS: progression-free survival
PPES: palmar-plantar erythrodysesthesia syndrome
TRAE: treatment-related adverse events
UC: urothelial carcinoma
