# Supplementary material for: Efficacy and safety of first- versus second-generation fibroblast growth factor receptor (FGFR) inhibitors in advanced or metastatic urothelial carcinoma with FGFR alterations: a systematic review and meta-analysis
Source: Front Pharmacol. 2026 Sep 30;17:1850131. doi: 10.3389/fphar.2026.1850131 (PMC13662917; doi:10.3389/fphar.2026.1850131)
Supplement: Supplementary file 1 [file Supplementaryfile1.docx]

Supplementary Table 1 The search strategy in PubMed, Web of Science and Embase databases on July 10 2025.

| Databases | Search | Query | Results |
| --- | --- | --- | --- |
| PubMed | #1 | (((((((((((((((((fibroblast growth factor receptor[Title/Abstract]) OR (FGFR[Title/Abstract])) OR (erdafitinib[Title/Abstract])) OR (JNJ-42756493[Title/Abstract])) OR (infigratinib[Title/Abstract])) OR (BGJ398[Title/Abstract])) OR (pemigatinib[Title/Abstract])) OR (INCB054828[Title/Abstract])) OR (derazantinib[Title/Abstract])) OR (ARQ-087[Title/Abstract])) OR (rogaratinib[Title/Abstract])) OR (BAY 1163877[Title/Abstract])) OR (futibatinib[Title/Abstract])) OR (Lytgobi[Title/Abstract])) OR (dovitinib[Title/Abstract])) OR (TKI258[Title/Abstract])) OR (fexagratinib[Title/Abstract])) OR (AZD4547[Title/Abstract]) | 9,977 |
|  | #2 | **(((urothelial carcinoma[Title/Abstract]) OR (urothelial cancer[Title/Abstract])) OR (bladder cancer[Title/Abstract])) OR (urinary bladder[Title/Abstract])** | 85,379 |
|  | #3 | #1 AND #2 | 648 |
| Web of Science | #1 | TS=(fibroblast growth factor receptor) OR TS=(FGFR) OR TS=(erdafitinib) OR TS=(JNJ-42756493) OR TS=(infigratinib) AND TS=(BGJ398) OR TS=(pemigatinib) OR TS=(INCB054828) OR TS=(derazantinib) OR TS=(ARQ-087) OR TS=(rogaratinib) OR TS=(BAY 1163877) OR TS=(futibatinib) OR TS=(Lytgobi) OR TS=(dovitinib) OR TS=(TKI258) OR TS=(fexagratinib) OR TS=(AZD4547) | 44,427 |
|  | #2 | TS=(urothelial carcinoma) OR TS=(urothelial cancer) OR TS=(bladder cancer ) OR TS=(urinary bladder) | 162,143 |
|  | #3 | #1 AND #2 | 1,187 |
| Embase | #1 | 'fibroblast growth factor receptor':ab,ti OR fgfr:ab,ti OR erdafitinib:ab,ti OR 'jnj 42756493':ab,ti OR infigratinib:ab,ti OR bgj398:ab,ti OR pemigatinib:ab,ti OR incb054828:ab,ti OR derazantinib:ab,ti OR 'arq 087':ab,ti OR rogaratinib:ab,ti OR 'bay 1163877':ab,ti OR futibatinib:ab,ti OR lytgobi:ab,ti OR dovitinib:ab,ti OR tki258:ab,ti OR fexagratinib:ab,ti OR azd4547:ab,ti | 14,777 |
|  | #2 | 'urothelial cancer':ab,ti OR 'urothelial carcinoma':ab,ti OR 'bladder cancer':ab,ti OR 'urinary bladder':ab,ti | 120,311 |
|  | #3 | #1 AND #2 | 1210 |

Supplementary Table 2 Demographic and clinical characteristics of the patients at baseline.

| Study | Median age (range) | Sex  male (%) | ECOG | Primary tumor location  number (%) | FGFR alterations | Previous lines of systemic therapy |
| --- | --- | --- | --- | --- | --- | --- |
| Loriot2023 | 66 (32–85) | 96 (70.6) | 0-2 | upper tract 41 (30.1);  lower tract 95 (69.9) | FGFR3/2 | 1-3 |
| Siefker-Radtke2023（III） | 67 (44-86) | 142 (81.1) | 0-2 | upper tract 42 (24.0);  lower tract 133 (76.0) | FGFR3/2 | 1-2 |
| Siefker-Radtke2022 | 67 (61–73) | NA | 0-2 | upper tract 25 (25%);  lower tract 76 (75%) | FGFR3/2 | 0-3+ |
| Siefker-Radtke2023（II） | 72 | 33(76.7) | NA | NA | FGFR3/2 | 0 |
| Soria2016 | NA | NA | 0-1 | NA | FGFR | NA |
| Jiang2024 | 70 | 29 (79) | NA | NA | FGFR3/2 | NA |
| Rouvinov2023 | 73 | 16（64） | 0-2 | upper tract 14 (56%);  lower tract 11 (44%) | FGFR3/2 | 0-3 |
| Monteiro2021 | 76 (57–83) | 9 (75) | NA | NA | FGFR3/2 | 1-3 |
| Guercio2021 | NA | NA | NA | NA | FGFR3/2 | NA |
| Sternberg2022 | 69 (36-82) | 75 (86.2) | 0-1 | upper tract 29 (33.3%);  lower tract 58(66.7%) | FGFR1/3 | 1+ |
| Sweis2024 | 75（47-85） | 32(87) | 0-1 | upper tract 13 (35%);  lower tract 24(65%) | FGFR1/3 | NA |
| Joerger2018 | NA | NA | NA | NA | FGFR3/2/1 | NA |
| Necchi2023 | 68 (38-92) | 192 (73.8) | 0-2 | upper tract 105 (40.4%);  lower tract 173 (66.5) | FGFR3 | 0-2+ |
| Koshkin2024 | 73 | 37(86) | 0-1 | NA | FGFR3/2/1 | 0 |
| Pal2018 | 67 ( 39–85) | 46 (68.7) | 0-2 | NA | FGFR3 | 0-2+ |
| Milowsky2014 | 67 (46–81) | 33 (75) | 0-2 | NA | FGFR3 | NA |
| Necchi2024 | 68 (31-88) | 34 (69.4) | 0-2 | upper tract 35 (71.4%);  lower tract 13 (26.5%)； | FGFR3/2/1 | 1-3+ |

Supplementary Table 3 The MINORS item score for non-randomized studies.

| Study | Q1 | Q2 | Q3 | Q4 | Q5 | Q6 | Q7 | Q8 | Score |
| --- | --- | --- | --- | --- | --- | --- | --- | --- | --- |
| Siefker-Radtke2022 | 2 | 2 | 2 | 2 | 2 | 2 | 2 | 2 | 16 |
| Siefker-Radtke（II）2023 | 2 | 2 | 2 | 2 | 0 | 2 | 2 | 0 | 12 |
| Soria2016 | 2 | 2 | 2 | 2 | 0 | 2 | 2 | 0 | 12 |
| Jiang2024 | 2 | 2 | 2 | 2 | 0 | 2 | 2 | 0 | 12 |
| Rouvinov2023 | 2 | 2 | 2 | 2 | 0 | 2 | 2 | 0 | 12 |
| Monteiro2021 | 2 | 2 | 2 | 2 | 0 | 2 | 2 | 0 | 12 |
| Guercio2021 | 2 | 0 | 2 | 2 | 0 | 2 | 2 | 0 | 10 |
| Sweis2024 | 2 | 2 | 2 | 2 | 0 | 2 | 2 | 0 | 12 |
| Joerger2018 | 2 | 0 | 2 | 2 | 0 | 2 | 2 | 0 | 10 |
| Necchi2023 | 2 | 2 | 2 | 2 | 0 | 2 | 2 | 0 | 12 |
| Koshkin2024 | 2 | 0 | 2 | 2 | 0 | 2 | 2 | 0 | 10 |
| Pal2018 | 2 | 2 | 2 | 2 | 0 | 2 | 2 | 0 | 12 |
| Milowsky2014 | 2 | 2 | 2 | 2 | 0 | 2 | 2 | 0 | 12 |
| Necchi2024 | 2 | 2 | 2 | 2 | 0 | 2 | 2 | 2 | 14 |

The items are scored 0 (not reported), 1 (reported but inadequate) or 2 (reported and adequate).

Supplementary Table 4 The survival rate of UC patients treated with FGFR inhibitors.

| Study | Intervention | Overall Survival | Progression-Free Survival |
| --- | --- | --- | --- |
| Loriot2023 | erdafitinib | 6 months: 85%  (95%CI 77- 90)  12 months: 51%  (95%CI 41 - 60) |  |
| Siefker-Radtke2023(III) | erdafitinib | 6 months: 77%  (95%CI 70 - 83)  12 months: 46%  (95%CI 39 - 54) |  |
| Siefker-Radtke2022 | erdafitinib | 12 months: 49 %  (95%CI 39 - 59)  24 months: 31%  (95%CI 22 - 40) | 12 months: 21%  (95%CI 13 - 29) |
| Sweis2024 | rogaratinib+ atezolizumab | 24 months: 31% | 24 months: 27% |
| Koshkin2024 | futibatinib+  pembrolizumab | 12-month: 62% | 12 months: 49% |

Supplementary Table 5 The subgroup analysis of any grade TRAEs in first- and second-generation FGFR inhibitors.

| Subgroup | Numbers | Pooled rate of any grade TRAEs (95% CI ) | Heterogeneity | | Significance test *p* |
| --- | --- | --- | --- | --- | --- |
|  |  |  | I^2^ | P value |  |
| First-/second-geranation FGFR inhibitors | | | | | |
| first-generation FGFR inhibitors | 2 | 0.98 (95%CI 0.94 to 1.00 ) | NA | NA | 0.666 |
| second-generation FGFR inhibitors | 10 | 0.97 (95%CI 0.96 to 0.99) | 29.22% | 0.18 |  |
| Selective FGFR inhibitors | | | | | |
| pan-FGFR inhibitors | 8 | 0.98 (95%CI 0.96 to 0.99) | 21.48% | 0.26 | 0.274 |
| FGFR1/2/3 inhibitors | 2 | 0.95 (95%CI 0.93 to 0.98) | NA | NA |  |
| Therapeutic regimen | | | | | |
| erdafitinib monotherapy | 5 | 0.98 (95%CI 0.96 to 1.00) | 27.88% | 0.24 | 0.294 |
| rogaratinib monotherapy | 1 | 0.94 (95%CI 0.87 to0.98) | NA | NA |  |
| rogaratinib/futibatinib combined with immunotherapy | 2 | 0.97 (95%CI 0.89 to 1.00) | NA | NA |  |

Supplementary Table 6 The subgroup analysis of grade ≥3 TRAEs in first- and second-generation FGFR inhibitors.

| Subgroup | Numbers | Pooled rate of grade ≥3 TRAEs (95% CI ) | Heterogeneity | | Significance test *p* |
| --- | --- | --- | --- | --- | --- |
|  |  |  | I^2^ | P value |  |
| First/second generation FGFR inhibitors | | | | | |
| first-generation FGFR inhibitors | 2 | 0.67(95% CI 0.57to 0.76 ) | NA | NA | **0.006** |
| second-generation FGFR inhibitors | 10 | 0.49(95% CI0.40 to 0.57) | 80.75% | <0.01 |  |
| Selective FGFR inhibitors | | | | | |
| pan-FGFR inhibitors | 8 | 0.47(95%CI0.37 to 0.56) | 77.59% | <0.01 | 0.913 |
| FGFR1/2/3 inhibitors | 2 | 0.47(95%CI0.42 to 0.53) | NA | NA |  |
| Therapeutic regimen | | | | | |
| monotherapy | 8 | 0.50(95%CI0.41to 0.59) | 94.23% | <0.01 | 0.179 |
| Combined with immunotherapy | 2 | 0.39(95%CI0.26 to 0.52) | NA | NA |  |
| Pan-FGFR inhibitors | | | | | |
| erdafitinib monotherapy | 5 | 0.51(95%CI0.38 to 0.64) | 82.97% | <0.01 | 0.224 |
| rogaratinib monotherapy | 1 | 0.37(95%CI0.27 to0.48) | NA | NA |  |
| rogaratinib/futibatinib combined with immunotherapy^*^ | 2 | 0.39(95%CI0.26 to 0.52) | NA | NA |  |

Supplementary Table 7 The subgroup analysis of any grade hyperphosphatemia in first- and second-generation FGFR inhibitors.

| Subgroup | Numbers | Pooled rate of any grade hyperphosphatemia (95% CI ) | Heterogeneity | | Significance test *p* |
| --- | --- | --- | --- | --- | --- |
|  |  |  | I^2^ | P value |  |
| First/second generation FGFR inhibitors | | | | | |
| first-generation FGFR inhibitors | 2 | 0.06(95% CI 0.02to 0.12 ) | NA | NA | <0.001 |
| second-generation FGFR inhibitors | 12 | 0.60(95% CI0.49 to 0.70) | 90.56% | <0.01 |  |
| Selective FGFR inhibitors | | | | | |
| pan-FGFR inhibitors | 10 | 0.63(95%CI0.53 to 0.73) | 85.12% | <0.01 | 0.001 |
| FGFR1/2/3 inhibitors | 2 | 0.43(95%CI0.38 to 0.49) | NA | NA |  |
| pan-FGFR inhibitors | | | | | |
| erdafitinib | 7 | 0.71(95%CI0.62 to 0.79) | 69.86% | <0.01 | <0.001 |
| rogaratinib | 2 | 0.45(95%CI0.37 to0.54) | NA | NA |  |
| futibatinib | 1 | 0.53(95%CI0.38 to 0.69) | NA | NA |  |

Supplementary Table 8 The subgroup analysis of grade ≥3 hyperphosphatemia in first- and second-generation FGFR inhibitors.

| Subgroup | Numbers | Pooled rate of grade ≥3 hyperphosphatemia (95% CI ) | Heterogeneity | | Significance test *p* |
| --- | --- | --- | --- | --- | --- |
|  |  |  | I^2^ | P value |  |
| First/second generation FGFR inhibitors | | | | | |
| first-generation FGFR inhibitors | 2 | 0.01(95% CI 0.00 to 0.03) | NA | NA | 0.017 |
| second-generation FGFR inhibitors | 12 | 0.00(95% CI 0.00 to 0.02 ) | 52.19% | 0.02 |  |
| Selective FGFR inhibitors | | | | | |
| pan-FGFR inhibitors | 10 | 0.02(95%CI 0.00 to 0.04) | 56.61% | 0.01 | 0.161 |
| FGFR1/2/3 inhibitors | 2 | 0.01(95%CI0.00 to 0.02) | NA | NA |  |
| pan-FGFR inhibitors | | | | | |
| erdafitinib | 7 | 0.02(95%CI 0.00 to 0.04) | 39.41% | 0.13 | 0.004 |
| rogaratinib | 2 | 0.00(95%CI0.00 to0.01) | NA | NA |  |
| futibatinib | 1 | 0.18(95%CI0.04to 0.43) | NA | NA |  |

Supplementary Table 9 The subgroup analysis of any grade diarrhea in first- and second-generation FGFR inhibitors.

| Subgroup | Numbers | Pooled rate of any grade diarrhea (95% CI ) | Heterogeneity | | Significance test *p* |
| --- | --- | --- | --- | --- | --- |
|  |  |  | I^2^ | P value |  |
| First/second geranation FGFR inhibitors | | | | | |
| first-generation FGFR inhibitors | 2 | 0.45(95% CI 0.35to 0.55 ) | NA | NA | 0.302 |
| second-generation FGFR inhibitors | 12 | 0.37(95% CI0.28 to 0.48) | 88.73% | <0.001 |  |
| Selective FGFR inhibitors | | | | | |
| pan-FGFR inhibitors | 10 | 0.38(95%CI0.27 to 0.50) | 88.57% | <0.001 | 0.938 |
| FGFR1/2/3 inhibitors | 2 | 0.39(95%CI0.34 to 0.44) | NA | NA |  |
| Pan-FGFR inhibitors | | | | | |
| erdafitinib | 7 | 0.32(95%CI0.18 to 0.49) | 91.86% | <0.001 | 0.117 |
| rogaratinib | 2 | 0.52(95%CI0.43 to0.61) | NA | NA |  |
| futibatinib | 1 | 0.44(95%CI0.29 to 0.60) | NA | NA |  |

Supplementary Table 10 The subgroup analysis of grade ≥3 diarrhea in first- and second-generation FGFR inhibitors.

| Subgroup | Numbers | Pooled rate of any grade diarrhea (95% CI ) | Heterogeneity | | Significance test *p* |
| --- | --- | --- | --- | --- | --- |
|  |  |  | I^2^ | P value |  |
| First/second geranation FGFR inhibitors | | | | | |
| first-generation FGFR inhibitors | 2 | 0.02(95% CI 0.00 to 0.06 ) | NA | NA | 0.482 |
| second-generation FGFR inhibitors | 12 | 0.03(95% CI0.02 to 0.05) | 0% | 0.61 |  |
| Selective FGFR inhibitors | | | | | |
| pan-FGFR inhibitors | 10 | 0.03(95%CI0.02 to 0.05) | 0% | 0.45 | 0.694 |
| FGFR1/2/3 inhibitors | 2 | 0.04(95%CI0.02 to 0.06) | NA | NA |  |
| Pan-FGFR inhibitors | | | | | |
| erdafitinib | 7 | 0.03(95%CI0.02 to 0.06) | 17.73% | 0.29 | 0.439 |
| rogaratinib | 2 | 0.02(95%CI0.00 to0.06) | NA | NA |  |
| futibatinib | 1 | 0.44(95%CI0.29 to 0.60) | NA | NA |  |

Supplementary Table 11 The subgroup analysis of any grade stomatitis in first- and second-generation FGFR inhibitors.

| Subgroup | Numbers | Pooled rate of any grade stomatitis (95% CI ) | Heterogeneity | | Significance test *p* |
| --- | --- | --- | --- | --- | --- |
|  |  |  | I^2^ | P value |  |
| First/second generation FGFR inhibitors | | | | | |
| first-generation FGFR inhibitors | 1 | 0.04(95% CI 0.00 to 0.14 ) | NA | NA | **<0.001** |
| second-generation FGFR inhibitors | 10 | 0.33(95% CI0.23 to 0.43) | 89.9% | <0.001 |  |
| Selective FGFR inhibitors | | | | | |
| pan-FGFR inhibitors | 8 | 0.32(95%CI0.19 to 0.46) | 91.47% | <0.001 | 0.377 |
| FGFR1/2/3 inhibitors | 2 | 0.39(95%CI0.34 to 0.44) | NA | NA |  |
| Pan-FGFR inhibitors | | | | | |
| erdafitinib | 6 | 0.44(95%CI0.35 to 0.53) | 65.27% | 0.01 | **<0.001** |
| rogaratinib | 2 | 0.10(95%CI0.05 to0.17) | NA | NA |  |

Supplementary Table 12 The subgroup analysis of ≥3 grade stomatitis in first- and second-generation FGFR inhibitors.

| Subgroup | Numbers | Pooled rate of ≥3 grade stomatitis (95% CI ) | Heterogeneity | | Significance test *p* |
| --- | --- | --- | --- | --- | --- |
|  |  |  | I^2^ | P value |  |
| First/second generation FGFR inhibitors | | | | | |
| first-generation FGFR inhibitors | 1 | 0.00(95% CI 0.00 to 0.07) | NA | NA | **<0.001** |
| second-generation FGFR inhibitors | 11 | 0.07(95% CI0.04 to 0.10) | 54.26% | 0.02 |  |
| Selective FGFR inhibitors | | | | | |
| pan-FGFR inhibitors | 9 | 0.07(95%CI0.04 to 0.12) | 57.83% | 0.02 | 0.741 |
| FGFR1/2/3 inhibitors | 2 | 0.07(95%CI0.05 to 0.10) | NA | NA |  |
| Pan-FGFR inhibitors | | | | | |
| erdafitinib | 7 | 0.09(95%CI0.07 to 0.12) | 0% | 0.48 | **0.001** |
| rogaratinib | 2 | 0.01(95%CI0.00 to0.05) | NA | NA |  |

Supplementary Table 13 The subgroup analysis of any grade PPES in second-generation FGFR inhibitors.

| Subgroup | Numbers | Pooled rate of any grade PPES (95% CI ) | Heterogeneity | | Significance test *p* |
| --- | --- | --- | --- | --- | --- |
|  |  |  | I^2^ | P value |  |
| erdafitinib | 5 | 0.25(95%CI0.20 to 0.31) | 47.01% | 0.11 | **0.014** |
| rogaratinib | 1 | 0.11(95%CI0.03 to0.25) | NA | NA |  |
| infigratinib | 1 | 0.12(95%CI0.05 to0.22) | NA | NA |  |

Supplementary Table 14 The subgroup analysis of ≥3 grade PPES in second-generation FGFR inhibitors.

| Subgroup | Numbers | Pooled rate of any grade PPES (95% CI ) | Heterogeneity | | Significance test *p* |
| --- | --- | --- | --- | --- | --- |
|  |  |  | I^2^ | P value |  |
| erdafitinib | 6 | 0.07(95%CI0.04 to 0.11) | 33.71% | 0.18 | 0.554 |
| rogaratinib | 1 | 0.03(95%CI0.00 to0.14) | NA | NA |  |
| infigratinib | 1 | 0.07(95%CI0.02 to0.17) | NA | NA |  |

Supplementary Table 15 The P values of Begg’s and Egger’s test for publication bias.

| Tests for publication bias | P value | | | | |
| --- | --- | --- | --- | --- | --- |
|  | ORR | DCR | any grade TRAEs | grade ≥3 TRAEs | adverse events leading to treatment discontinuation |
| Begg’s test | 0.837 | 0.310 | 0.583 | 0.583 | 0.754 |
| Egger’s test | 0.808 | 0.082 | 0.967 | 0.485 | 0.987 |

Records identified from:

PubMed (n =648)

Web of Science (n=1187)

Embase (n=1210)

manual search (n=3)

Records removed before screening (n =2521)

1.duplicate records

2. review, case report, editorial letter，meta-analysis

**Identification**

Records excluded (n =260)

1. not related to urothelial carcinoma
2. not related to FGFR inhibitors

Records screened

(n =527)

Reports not retrieved (n = 245)

1. not related to the efficacy or safety of FGFR inhibitors
2. not reporting of primary outcomes
3. lack of necessary information

Reports sought for retrieval

(n = 267)

**Screening**

Reports excluded (n = 5)

1. subgroup analysis of the same study
2. preliminary results or long-term follow-up of the same study

Reports assessed for eligibility

(n =22)

Studies included in review

(n =17)

**Included**

Supplementary Figure 1 PRISMA Flow diagram for included studies’ selection.

Supplementary Figure 2 Traffic light plots and bar plots for risk of bias assessment in randomized controlled trials.

Supplementary Figure 3 Forest plots for OS of FGFR inhibitors compared to chemotherapy or immunotherapy.

Supplementary Figure 4 Forest plots for PFS of FGFR inhibitors compared to chemotherapy or immunotherapy.

Supplementary Figure 5 Sensitivity analysis for ORR of different FGFR inhibitors.

Supplementary Figure 6 Sensitivity analysis for DCR of different FGFR inhibitors.

Supplementary Figure 7 Sensitivity analysis for incidence of any grade TRAEs.

Supplementary Figure 8 Sensitivity analysis for incidence of grade ≥3 TRAEs.

Supplementary Figure 9 Sensitivity analysis diagram for incidence of adverse events leading to treatment discontinuation.

Supplementary Figure 10 Funnel plot for ORR of different FGFR inhibitors.

Supplementary Figure 11 Funnel plot for DCR of different FGFR inhibitors.

Supplementary Figure 12 Funnel plot for incidence of any grade TRAEs.

Supplementary Figure 13 Funnel plot for incidence of grade ≥3 TRAEs.

Supplementary Figure 14 Funnel plot for incidence of adverse events leading to treatment discontinuation.
